# Quantitative perfusion imaging of neoplastic liver lesions: A multi-institution study

**DOI:** 10.1038/s41598-018-20726-1

**Published:** 2018-03-21

**Authors:** Shivani Pahwa, Hao Liu, Yong Chen, Sara Dastmalchian, Gregory O’Connor, Ziang Lu, Chaitra Badve, Alice Yu, Katherine Wright, Hamid Chalian, Shengxiang Rao, Caixia Fu, Ignacio Vallines, Mark Griswold, Nicole Seiberlich, Mengsu Zeng, Vikas Gulani

**Affiliations:** 1 Radiology, Case Western Reserve University, Cleveland, OH United States; 2 Radiology, Zhongshan Hospital, Fudan University, Shanghai, China; 3 Radiology, University Hospitals, Cleveland, OH United States; 4 Siemens Shenzhen Magnetic Resonance Ltd, Shenzhen, China; 5 MR Collaboration NE Asia, Siemens Healthcare, Shanghai, China; 6 Biomedical Engineering, Case Western Reserve University, Cleveland, OH United States

## Abstract

We describe multi-institutional experience using free-breathing, 3D Spiral GRAPPA-based quantitative perfusion MRI in characterizing neoplastic liver masses. 45 patients (age: 48–72 years) were prospectively recruited at University Hospitals, Cleveland, USA on a 3 Tesla (T) MRI, and at Zhongshan Hospital, Shanghai, China on a 1.5 T MRI. Contrast-enhanced volumetric T1-weighted images were acquired and a dual-input single-compartment model used to derive arterial fraction (AF), distribution volume (DV) and mean transit time (MTT) for the lesions and normal parenchyma. The measurements were compared using two-tailed Student’s t-test, with Bonferroni correction applied for multiple-comparison testing. 28 hepatocellular carcinoma (HCC) and 17 metastatic lesions were evaluated. No significant difference was noted in perfusion parameters of normal liver parenchyma and neoplastic masses at two centers (p = 0.62 for AF, 0.015 for DV, 0.42 for MTT for HCC, p = 0.13 for AF, 0.97 for DV, 0.78 for MTT for metastases). There was statistically significant difference in AF, DV, and MTT of metastases and AF and DV of HCC compared to normal liver parenchyma (p < 0.5/9 = 0.0055). A statistically significant difference was noted in the MTT of metastases compared to hepatocellular carcinoma (p < 0.001*10-5). In conclusion, 3D Spiral-GRAPPA enabled quantitative free-breathing perfusion MRI exam provides robust perfusion parameters.

## Introduction

The role of qualitative multiphasic MRI is well established in characterizing focal liver lesions as solid or cystic, characterising lesions with classical imaging patterns such as hepatocellular carcinoma, and distinguishing these from metastases, infectious lesions etc^1–3^. Conventional imaging biomarkers including tumor size, enhancement, and TNM stage have played a crucial role in clinical decision making. However, with the advent of newer oncologic drugs that disrupt fundamental growth mechanisms such as angiogenesis, the conventional imaging biomarkers are insufficient for tumor evaluation and follow up^4,5^. Moreover, approaching an era of personalized medicine, it is increasingly desirable to develop non-invasive, imaging-based metrics that detect phenotypic signatures of tumors a priori in order to select the optimal treatment regimen for a given patient^6^. This would help avoid unnecessary toxic effects of ineffective chemotherapeutic regimens.

Recent advances in perfusion MRI techniques enable volumetric coverage of the liver with a high spatial and temporal resolution^7–11^. Capturing the passage of contrast through the liver parenchyma and tumor using ultrafast free breathing MRI techniques enables extraction of tissue properties such as blood flow, extracellular space, time to peak, permeability etc^12^. These ultrafast MRI techniques offer distinct advantages over some earlier perfusion MRI methods in which data were acquired in breath holds or shared across frames^13,14^, which limited the quantitative perfusion evaluation to either a semi-quantitative analysis performed using signal intensity curves or resulted in perfusion parameters potentially corrupted by motion or poor temporal data fidelity^15–18^. Multiple clinical trials have used perfusion MRI based biomarkers to evaluate the response of hepatocellular carcinoma and hepatic metastases antivascular therapies^19–23^. However, these parameters suffer from methodological pitfalls as described above and have not been tested or applied outside academic centers. Moreover, the same method has been shown to give variable results with different MRI scanners and significant variations have been noted between patients and even between the same patient^4^.

Recently, a free breathing, 3-dimensional Through-time Spiral Generalized Autocalibrating Partially Parallel Acquisition Acceleration (GRAPPA) sequence was described, which enabled 3D coverage of the whole liver with a high spatiotemporal resolution, and also provided robust perfusion parameters while maintaining acceptable clinical image quality^9^. Here, initial clinical experience with this technique is described, for evaluating neoplastic liver masses in a multi-institutional study in the USA and China, at two different field strengths. We also attempt to further characterize the metastatic lesions according to the tissue of origin using perfusion parameters.

## Materials and Methods

The datasets generated and/or analyzed during the current study are available from the corresponding author on reasonable request.

This prospective study was conducted with the approval of the University Hospitals institutional review board and the ethics committee of Zhongshan Hospital, Fudan University, and is compliant with the Health Insurance Portability and Accountability Act. Written, informed consent was obtained from patients and volunteers. 10 asymptomatic normal volunteers (male-female ratio, 7:3; age, 20.9 ± 1.3 years) were recruited from March 2013 through April 2014 at the University Hospitals, USA in order to establish control perfusion parameters in normal liver parenchyma. Patients with focal liver lesions who underwent MRI examination as part of their routine clinical care at University Hospitals, Cleveland, USA and Zhongshan Hospital, Shanghai, China from March 2013 to August 2015 were recruited in the study. The inclusion criteria included patients within the age range 18–100 years with a known liver lesion, without a contra-indication for an MRI examination and GFR ≥ 40 ml/min/1.73 m^2^. The exclusion criteria included patients with contra-indications for MRI (claustrophobia, MRI incompatible medical devices, or embedded foreign bodies), pregnancy, kidney disease with GFR less 30 ml/mim/1.73 m^2^, minors and patients belonging to vulnerable population groups. Based on these criteria, 17 patients were recruited in the USA (age range 65.8+/− 6.8 years; male: female 11:4). 28 patients were recruited in China (age range 58.8+/− 10 years; male: female 20:11). The perfusion examination was performed separately from the clinical MRI examination (within 15 days) to avoid interfering with routine care. Image-guided biopsies were performed to establish the diagnosis of liver masses in all patients. No biopsy was performed for the incidentally encountered benign because the imaging findings were diagnostic.

### 3D image acquisition and reconstruction

The study was conducted at 3 T field strength in the USA (MAGNETOM Skyra, Siemens Healthcare, Erlangen, Germany) and 1.5 T field strength in China (MAGNETOM Aera, Siemens Healthcare, Erlangen, Germany), using 20–30 coil elements. Contrast-enhanced T1-weighted 3D volumes were acquired using an interleaved variable-density stack-of-spirals spoiled gradient echo sequence with fat suppression and a GRAPPA reduction factor of 6. 100 to 120 3D imaging volumes (time frames) were acquired in 4 minutes with a temporal resolution of 1.6 to 1.9 seconds per volume, and a spatial resolution of 1.9 × 1.9 × 3 mm3. 0.1 mmol/kg of gadobenate (Multihance; Bracco Diagnostics, Princeton, NJ) was injected after the fifth frame, at the rate of 3 mL/s, followed by 20 mL saline flush. A separate calibration scan of 3 fully sampled free breathing 3D volumes was acquired at the end of the perfusion examination to calculate GRAPPA weights for image reconstruction. Details of imaging parameters are provided in Table 1. The undersampled spiral images were reconstructed offline on a desktop computer (Intel Xeon E3-1270 quad-core CPUs at 3.4 GHz and 16GB of RAM) in MATLAB (The Mathworks, Natick, MA) using a previously described reconstruction algorithm^9^. To nullify the effects of respiratory motion and variation between frames due to changing contrast concentration, the reconstructed images were registered to their nearest temporal neighbor in a reference list^9^. This reference list was created by selecting multiple volumes from the same respiratory level using an edge detection method that measured the craniocaudal excursion of the liver. The time frames where liver is in the same position, which is approximately 20–25% of the total frames in each examination, formed a reference list; registration was performed using FMRIB’s Non-linear Image Registration Tool^24^.

**Table 1:** Imaging parameters for through time spiral GRAPPA perfusion MRI examination.

	DCE MRI
FOV*, mm	360–440
Matrix size	192–240
In-plane resolution, mm	1.9
TR/TE, milliseconds	4.5–4.7/0.6
Flip angle, degrees	15
Number of slices	60
Slice thickness, mm	3
Parallel imaging	6
Acquisition time per volume, seconds	1.6–1.9

^*^FOV – field of view.

### Perfusion quantification

The regions of interest (ROI) were drawn by a radiologist with 9 years of experience in abdominal imaging (SP). These were drawn on abdominal aorta and portal vein to measure arterial input function (AIF) and portal input function. Contrast concentration was derived from longitudinal relaxation time T1, which in turn was calculated from tissue SI using the signal equation for spoiled gradient echo^9^. Perfusion quantification was performed using a dual input, single compartment model described by Materne *et al*.^12^.

$$\frac{d{C}_{L}(t)}{dt}={k}_{1a}{C}_{a}(t-{\tau }_{a})+{k}_{1p}{C}_{p}(t-{\tau }_{b})-{k}_{2}{C}_{L}(t)$$

where C_L_(t), C_a_(t) and C_p_(t) represent the contrast concentration in tissue, aorta and portal vein, respectively; k_1a_ is the aortic inflow rate constant, k_1p_ is the portal venous inflow rate constant and k_2_ is the outflow rate constant; τ_a_ and τ_b_ are the transit time for aorta and portal vein, respectively. Perfusion maps were generated on a voxel-by-voxel basis and the following perfusion parameters were calculated: arterial fraction (AF = *k*_1a_/(*k*_1a_ + *k*_1p_)), distribution volume (DV = (*k*_1a_ + *k*_1p_)/*k*_2_) and mean transit time (MTT = 1/*k*_2_).

In normal volunteers, the ROIs were drawn in the right lobe of liver away from major vessels. In patients, the ROIs (mean ROI size – 69.9 voxels) were drawn on the enhancing part of the lesion as well as in the normal appearing liver parenchyma. In patients with hypovascular lesions, the ROI was simply drawn on the solid portion of the lesion.

### Morphologic evaluation

Image quality, lesion size, number and pattern of enhancement were evaluated by two independent radiologists with 15 (VG) and 8 (SP) years of experience in abdominal MRI. The spiral GRAPPA images were visualized as cine images that captured the passage of gadolinium through the live parenchyma for the duration of the scan. These were compared to the images obtained from the standard clinical post-contrast VIBE images in arterial, venous and delayed phases.

### Statistical analysis

Two tailed Student’s t-tests were used to compare the perfusion parameters between patient lesions and healthy volunteer normal liver. ANOVA test was used to determine the significance of difference in the perfusion parameters of metastases according to the tissue of origin. All results were corrected for multiple-comparison testing with the Bonferroni method. With this method, statistical significance was reached for P values < 0.05/k, where k represents the number of tests. To compare lesions between institutions, k was set to 6 (2 groups X 3 parameters) resulting in P < 0.0083. The comparison of normal parenchyma, HCC, and metastasis and the comparison of metastatic adenocarcinoma based on primary origin both set k to 9 (3 comparisons X 3 parameters) resulting in P < 0.0055.

## Results

**Figure 1:**
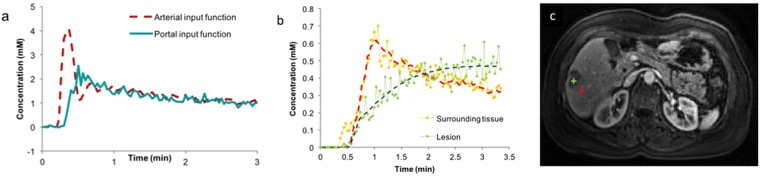
Perfusion modeling in a patient with metastatic breast cancer. Arterial and portal input function (**a**), concentration-time curve of the lesion and surrounding tissue (**b**) for the lesion depicted in T1-weighted image (**c**). Perfusion modeling revealed the lesion to have an arterial fraction of 95.3%, distribution volume of 48.0% and mean transit time of 96.6 seconds. The corresponding values for the liver parenchyma were arterial fraction of 16.9%, distribution volume of 30.8% and mean transit time of 5.2 seconds.

Clinically acceptable image quality was obtained and successful perfusion modeling was performed in all the patients and volunteers (Fig. 1). An example of arterial input function and portal input function is provided in Fig. 1a. The values of AF, DV and MTT in normal liver parenchyma in normal volunteers were 25 ± 4.3%, 29.4 ± 8.3% and 8.8 ± 6.1 seconds respectively (Table 2). In the USA, 5 patients were diagnosed with hepatocellular carcinoma and 10 patients with metastatic adenocarcinoma. In China, 23 patients were diagnosed with hepatocellular carcinoma and 7 patients with metastatic adenocarcinoma. A single representative lesion was evaluated in every patient and a total of 45 lesions were evaluated. In patients with metastases recruited in the USA, multiple lesions were evaluated in the patients to enrich the dataset in order to derive preliminary trends for perfusion parameters for metastatic lesions.

**Table 2:** Summary of comparisons between perfusion parameters between normal liver parenchyma, metastatic adenocarcinoma, and hepatocellular carcinoma.

	Arterial Fraction (%)	Distribution Volume (%)	Mean Transit Time (seconds)
Normal (n = 10)	25.0 ± 4.3	29.4 ± 8.3	8.8 ± 6.1
Metastatic Adenocarcinoma	79.1 ± 13.5	53.8 ± 25.0	41.3 ± 24.5
Metastatic vs. normal (P value)	6.37 × 10^−14^*	0.0004*	4.90 × 10^−05^*
Hepatocellular carcinoma	72.1 ± 14.5	49.0 ± 20.5	11.3 ± 4.8
HCC vs. normal (P value)	2.69 × 10–17*	0.0002*	0.196
Metastatic vs. HCC (P value)	0.0932	0.466	0.000116*

There was a statistically significant difference in AF, DV, and MTT^*^ of metastatic lesions and AF and DV of HCC compared to normal liver parenchyma in healthy volunteers (p < 0.5/9 = 0.0055). A statistically significant difference was noted in the MTT of metastases compared to hepatocellular carcinoma (p < 0.001*10^−5^). *P value < 0.05/9 = 0.0055 after Bonferroni correction for multiple-comparisons.

^*^AF - Arterial fraction; DV- Distribution volume; MTT – Mean transit time.

### Lesion morphology

**Figure 2:**
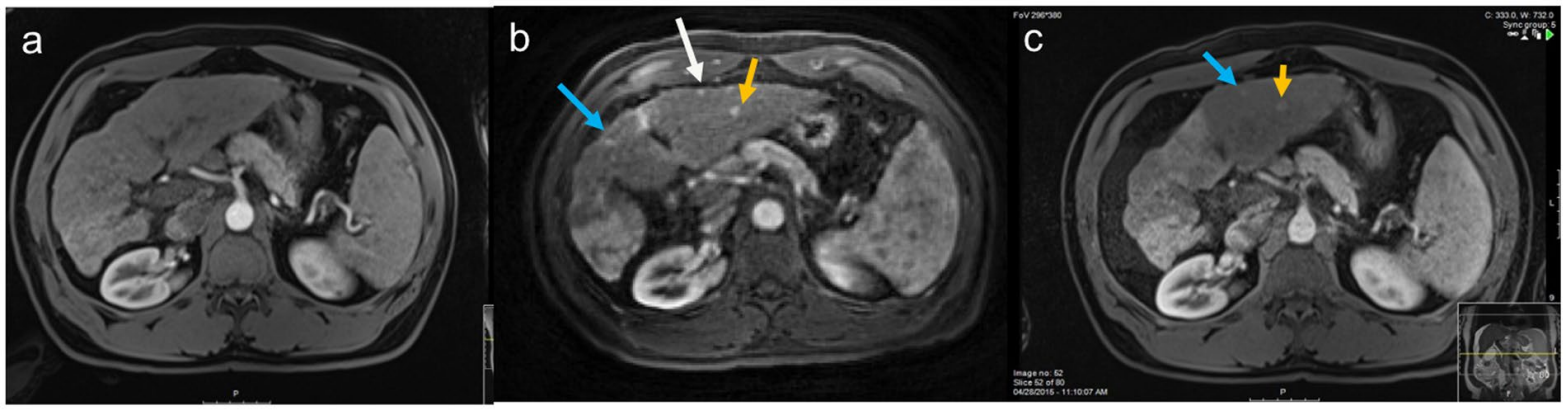
(**a**) Standard arterial phase VIBE image depicts an irregular liver contour in a patient with cirrhosis on surveillance for hepatocellular carcinoma- no focal lesion is seen. (**b**) Spiral DCE acquisition performed 12 days after the clinical study depicts three arterially enhancing lesions. These lesions were detected in the follow up clinical exam performed 3 months after the first exam (**c**).

The lesions ranged in size from 0.5–8.5 cm. The enhancement pattern of lesions observed in corresponding time points of the through time spiral GRAPPA acquisition was identical to that seen in clinical arterial, venous and delayed phase VIBEs. All lesions seen on clinical post-contrast VIBE images were seen on the spiral GRAPPA images. However, 3 small arterially enhancing lesions measuring approximately 0.7 cm in size that were seen on spiral GRAPPA images were not visualized in the clinical MRI examination performed 14 days earlier in a patient on active surveillance for hepatocellular carcinoma. These lesions were subsequently identified on the follow-up clinical MRI examination performed 3 months later (Fig. 2).

### Perfusion parameters for all neoplastic lesions

There was a statistically significant difference in AF and DV of malignant lesions compared to normal liver parenchyma in healthy volunteers (p < 0.05/9 = 0.0055). There was a statistically significant difference in AF and DV of HCC compared to normal liver parenchyma in healthy volunteers (p < 0.5/9 = 0.0055). There was no statistical difference in MTT of HCC compared to normal parenchyma in healthy volunteers (p = 0.196) A statistically significant difference was also noted in MTT of metastases compared to hepatocellular carcinoma (p < 0.05/9 = 0.0055). No significant difference was observed between the AF and DV of HCC and metastases (Table 3).

**Table 3:** Summary of Liver Perfusion Parameters in patients scanned in USA at 3 T field strength and in China at 1.5 T field strength.

	Site of MRI exam	Number of patients	Surrounding tissue			Lesion		
			Arterial fraction (%)	Distribution volume (%)	Mean transit time (sec)	Arterial fraction (%)	Distribution volume (%)	Mean transit time (sec)
Met	US	10	47.6 ± 12.2	34.7 ± 16.1	6.0 ± 3.5	83.1 ± 11.1*	53.9 ± 12.7*	42.8 ± 27.1*
	China	7	68.7 ± 6.3	27.4 ± 9.4	10.9 ± 2.3	73.9 ± 12.2	53.5 ± 31.5	39.2 ± 22.2*
	Mean	17	53.6 ± 14.5^#^	32.6 ± 14.5	7.4 ± 3.9	79.1 ± 13.5*	53.8 ± 25.0*	41.3 ± 24.5*
HCC	US	5	46.4 ± 22.6	34.9 ± 13.0	19.6 ± 9.2	75.0 ± 17.0*	36.0 ± 8.6	12.9 ± 3.6*
	China	23	57.5 ± 18.2	35.4 ± 6.9	16.8 ± 8.2	71.4 ± 14.2*	51.9 ± 21.3*	10.9 ± 5.0*
	Mean	28	54.8 ± 19.3^#^	35.3 ± 8.3	17.4 ± 8.3^#^	72.1 ± 14.5*	49.0 ± 20.5*	11.3 ± 4.8*

There was no significant difference in the perfusion parameters obtained for hepatocellular carcinoma and metastases at the two field strengths but there was significant difference in perfusion parameters between lesions and surrounding liver parenchyma. Met: Metastatic Adenocarcinoma.

HCC: Hepatocellular carcinoma.

*Represents significant difference (*P* < 0.05) between surrounding tissue and lesion.

^#^Represents significant difference (*P* < 0.05) between the apparently normal liver parenchyma. surrounding the lesions and the liver parenchyma in normal volunteers.

There was a statistically significant difference in the AF of the “lesion-free” liver parenchyma in patients with hepatocellular carcinoma and metastases (p < 0.05) compared to the lesions as well as the normal parenchyma in healthy volunteers. Additionally, there was a statistically significant difference the MTT of the “lesion-free” liver parenchyma in patients with hepatocellular carcinoma versus the lesions per se as well as the normal parenchyma in healthy volunteers (p < 0.05). There was no significant difference in MTT of “lesion-free” liver parenchyma in patients with metastases versus normal parenchyma in healthy volunteers. The distribution volume of the “lesion-free” liver parenchyma in patients versus lesions or normal parenchyma in healthy volunteers.

### Perfusion parameters: hepatocellular carcinoma

**Figure 3:**
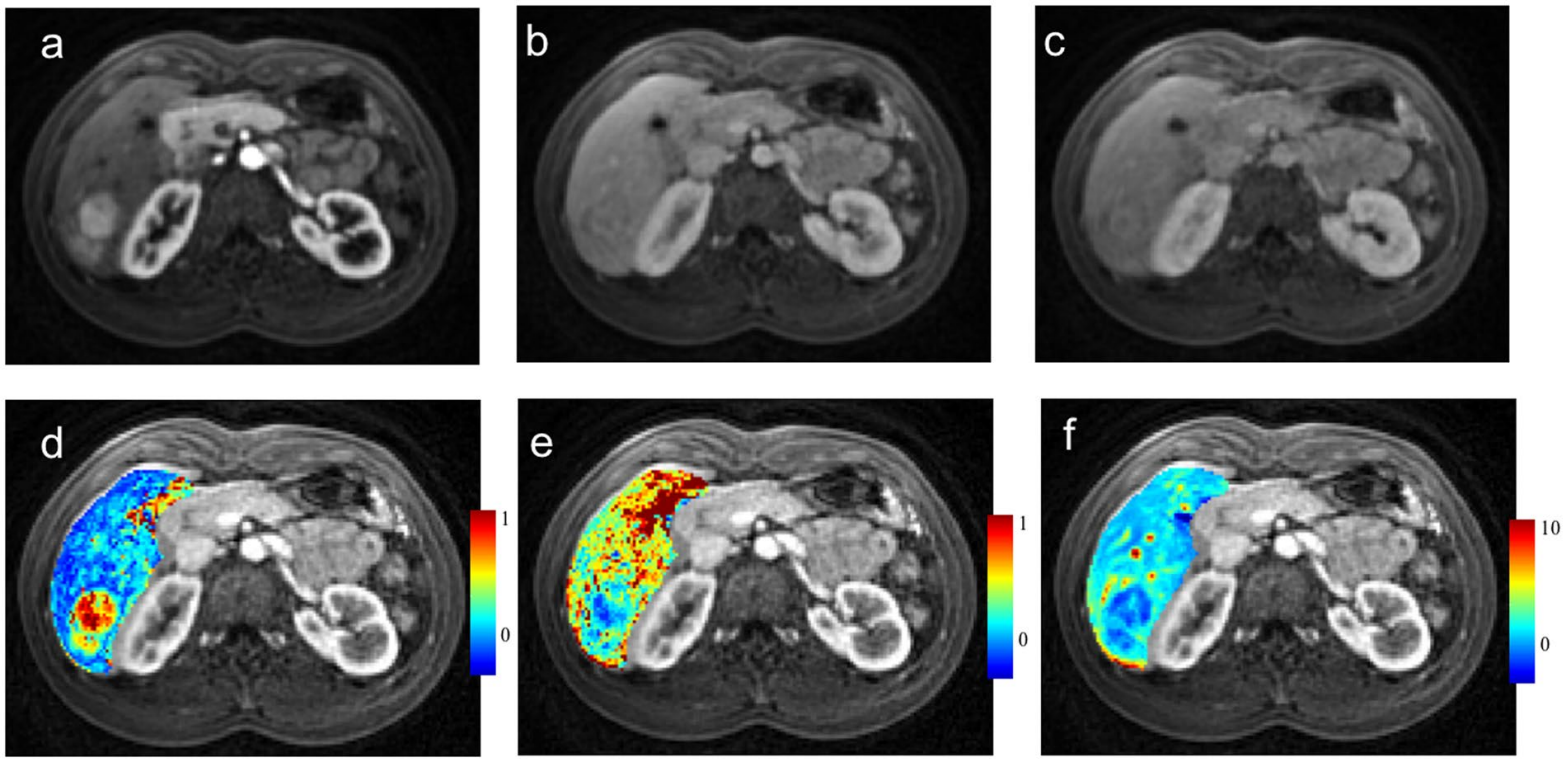
Liver perfusion maps for a patient with hepatocellular carcinoma. Free breathing spiral DCE images averaged over 9 frames show the lesion in arterial (**a**), venous (**b**) and delayed phases (**c**). Corresponding liver perfusion maps depict arterial fraction of 77% (**d**), distribution volume of 45% (**e**) and mean transit time of 12 seconds (**f**).

The perfusion parameters for hepatocellular carcinoma at 3 T field strength in the USA were as follows: AF was 75 ± 17%, DV was 36.0 ± 8.6% and MTT of 12.9 ± 3.6 seconds respectively (Figs 2, 3); the corresponding values were 71.4 ± 14.2%, 51.9% ± 21.3%, and 10.9 ± 5.0 seconds respectively at 1.5 T field strength machine in China. There was no statistical difference (P value < 0.05/6 = 0.0083) between the perfusion parameters obtained at 1.5 T and 3 T field strengths for these lesions after Bonferroni correction (p = 0.62 for AF, 0.015 for DV and 0.42 for MTT) (Table 3).

### Perfusion parameters: metastases

**Figure 4:**
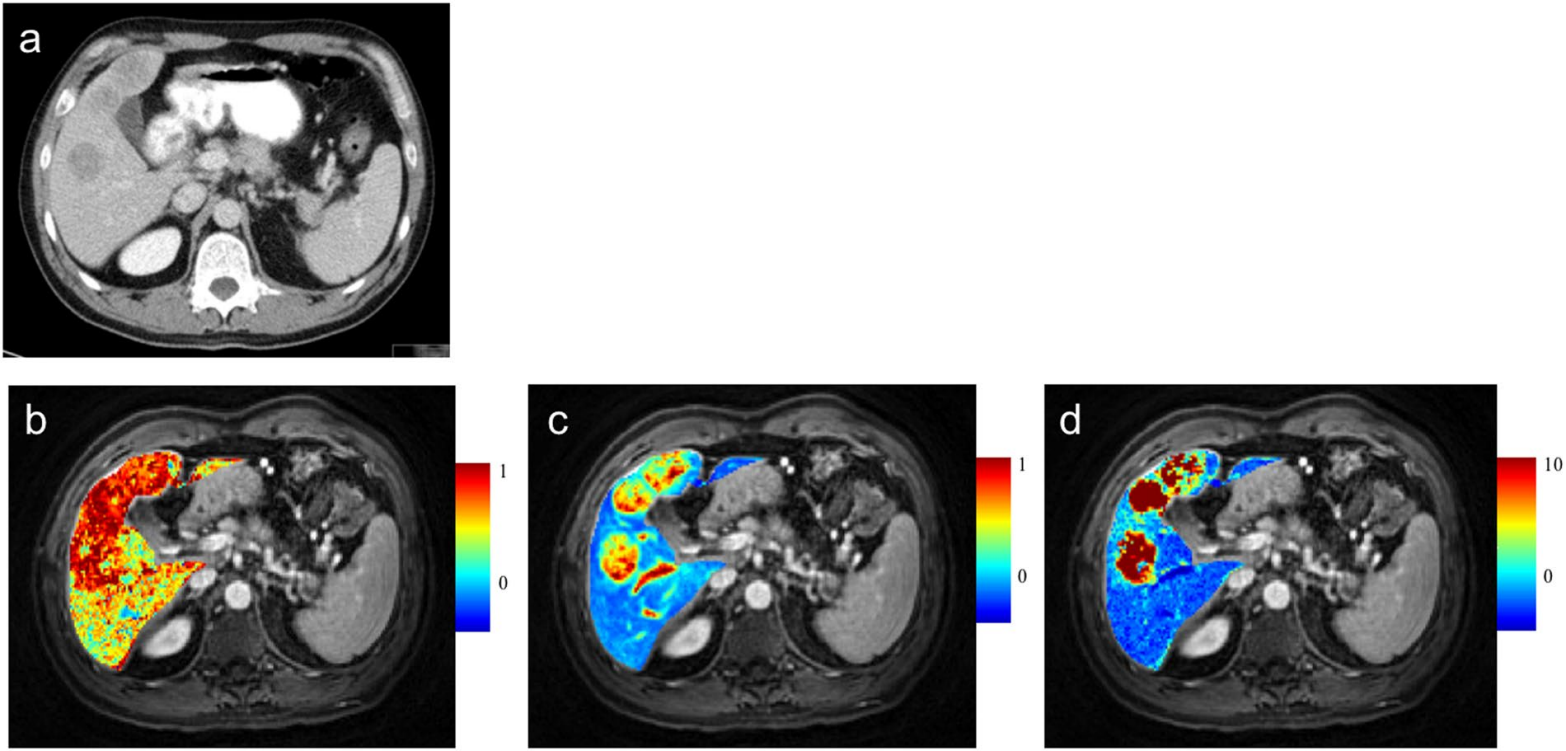
Liver perfusion maps for a patient with metastatic carcinoma lung. Axial CT image shows three hypodense lesions (**a**). Corresponding liver perfusion maps depict arterial fraction (**b**), distribution volume (**c**) and mean transit time (**d**).

The mean AF, DV and MTT for metastases were 83.1 ± 11.1%, 53.9% ± 12.7%, and 42.8 ± 27.1 seconds respectively at 3 T field strength magnet (Figs 1, 4). The corresponding values were 72.6 ± 11.8%, 54.5% ± 29.2%, and 38.9 ± 20.5 seconds respectively at 1.5 T field strength machine. Again, no statistically significant difference was seen between the parameters for these lesions at two field strengths (p = 0.13 for AF; 0.97 for DV and 0.78 for MTT) (Table 3).

Table 4 summarizes the mean perfusion parameters obtained for metastases according to the organ of origin. There was a statistically significant difference in the DV for metastatic lesions from breast vs colon (p = 0.0003) and metastatic lesions from colon vs lung (p < 0.0001).

**Table 4:** Summary of Perfusion parameters in metastases according to the organ of origin.

	Number of lesions	Arterial Fraction (%)	Distribution Volume (%)	Mean Transit Time (seconds)
Carcinoma breast	20	77.3 ± 17.0	62.5% ± 17.6%	40.6 ± 27.7
Colorectal cancer	20	64.3 ± 23.8%	41.4% ± 16.4%	50.6 ± 25.4
Carcinoma lung	6	81.5 ± 6.8%	66.4% ± 5.7%	37.5 ± 17.3

There was a statistically significant difference in the distribution volume (DV) for metastatic lesions from breast vs colon (p = 0.0003) and metastatic lesions from colon vs lung (p < 0.0001).

## Discussion

Materne *et al*. first described the derivation of hepatic perfusion parameters from a DCE MRI exam^12^. They described methods to derive hepatic arterial fraction (proportion of blood flow derived from hepatic artery), mean transit time, and distribution volume (corresponds to the volume of extracellular, extravascular space in a tissue which is a measure of the tissue cellularity). Measurement of these parameters has clinical utility because neoplastic liver lesions differ significantly from normal liver parenchyma in their blood supply, vascular permeability, tissue composition, and organization. However, measurement of these parameters entails capturing of the 3 to 5-minute journey of gadolinium through the liver. The commonly used clinical sequences require the patients to hold their breaths for image acquisition; breath-holds running into minutes required to obtain perfusion parameters are un-physiological. Hence, ultrafast, free breathing, quantitative MRI sequences are being developed and tested. However, the utility of these techniques in wider “real-world” clinical practice where the pressure on scanner through-put is high had not been tested.

The current study evaluated the robustness of free-breathing, 3D through-time spiral GRAPPA acquisition in two diverse tertiary care centers in the USA and China. The acquisition was designed to not require “tweaking” by the technologist. There was no statistically significant difference in perfusion parameters obtained for normal liver parenchyma and liver pathologies between the USA and China. This robustness of parameters is enabled by the fact that through-time spiral GRAPPA has a small temporal footprint of less than 2 seconds compared to view sharing techniques that have a broader temporal footprint of 3–4 seconds, which affects the accuracy of arterial input characterization. A smaller temporal resolution achieved with through time spiral GRAPPA acquisition allows accurate estimation of arterial and portal venous input functions without the need for rescaling^9^. To minimize the in-flow effects on arterial input function, ROI was drawn near the end of imaging slab as previously described^9^.

Our results showed that the AF and DV for HCC and metastases were significantly higher than in normal liver parenchyma. These results are in concordance with the fact that both primary and metastatic liver tumors derive their blood supply from the hepatic artery^25–28^. It has also been documented that an increase in the hepatic arterial fraction of blood supply of a malignant lesion correlates with increasing aggressive behavior^25,29,30^. Hence, reliable estimation of perfusion parameters could potentially help tailor chemotherapy regimens according to the tumor aggressiveness and potentially predict survival outcomes^29^.

Normal liver has large fenestrae that allow free exchange of contrast medium between the sinusoids and space of Disse and therefore has a low distribution volume^31^. On the other hand, both HCC and metastases have higher cellularity and restricted permeability resulting in a higher DV. Similar to the results obtained by Taouli *et al*.^32^, we did not find a significant difference in the MTT for HCC versus normal liver parenchyma. The MTT for metastases was significantly longer than that of HCC and normal liver parenchyma, similar to previous findings^27,33^. These have been attributed to the fact that metastatic cells get arrested in the liver microvasculature and compress hepatic sinusoids leading to an increased vascular resistance leading to an increased transit time^25,27,33^. Metastatic lesions are also more fibrotic compared to normal liver parenchyma. We also report the perfusion parameters for liver metastases from the colon, breast, and lung, which have not been reported in the literature and need further exploration.

All lesions seen on standard clinical examination were detected on the spiral perfusion MRI study with identical enhancement appearance at corresponding time points. Three sub-centimeter lesions which were not seen on the standard clinical arterial phase images in a patient on active surveillance for HCC, were detected with spiral GRAPPA DCE. While a single observation, this is not surprising as it has been reported that the standard acquisitions with a temporal resolution of 10–20 seconds have a sensitivity of only 4% for detection of sub-centimetric HCC^34^. Subtle differences in time of arterial enhancement in a lesion could easily result in non-detection of the lesion in current clinical studies, where single arterial time points are routinely acquired due to long acquisition times. Studies have shown that early detection of small HCC and institution of appropriate therapy greatly improves the prognosis for the patient^35^. Therefore, imaging that improves the sensitivity of early detection of such lesions could potentially improve therapeutic decisions.

We compared the perfusion parameters of the lesions with the apparently uninvolved liver parenchyma in the same patient. We found significant differences in the perfusion parameters of the lesions compared to the “lesion-free” parenchyma which is expected. However, we also found significant differences in the AF of lesion-free parenchyma in patients with metastases compared to parenchyma in normal volunteers. Studies have revealed that the seemingly normal parenchyma may harbor micro metastases which are beyond the resolution of available imaging methods but affect patient outcomes^36,37^. These can affect the vascularity of the surrounding parenchyma due to neoangiogenesis as well as mass effect. We found that AF and MTT of liver parenchyma in patients with hepatocellular carcinoma was altered compared to healthy volunteers. This could be related to the fact that these lesions arose in a background of cirrhosis. The cirrhotic liver parenchyma has been shown to have decreased portal venous perfusion, increased hepatic arterial flow and increased deposition of collagen in extracellular spaces leading to increased transit time^38^.

### Limitations

The number of patients recruited and lesions evaluated in this preliminary multicenter experience is small. Future studies that evaluate a wider spectrum of lesions in larger patient cohorts is desirable.

## Conclusions

This work shows that perfusion parameters obtained from quantitative modeling of a 3D free breathing through time spiral GRAPPA acquisition agree across institutions, continents and field strength. In addition to the comfort provided to both the patient and the technologist by a free breathing exam, the high temporal resolution made possible by such an approach enables a quantitative dimension to the contrast enhanced liver exam. These early results are encouraging and larger multi-center studies are required to further examine the value of perfusion MRI derived parameters as imaging biomarkers for management of hepatic neoplasms.
